# Upholding Tuberculosis Services during the 2014 Ebola Storm: An Encouraging Experience from Conakry, Guinea

**DOI:** 10.1371/journal.pone.0157296

**Published:** 2016-08-17

**Authors:** Nimer Ortuno-Gutierrez, Rony Zachariah, Desalegn Woldeyohannes, Adama Bangoura, Gba-Foromo Chérif, Francis Loua, Veerle Hermans, Katie Tayler-Smith, Welile Sikhondze, Lansana-Mady Camara

**Affiliations:** 1 Damien Foundation, Conakry, Guinea; 2 Medecins Sans Frontières, Medical department (Operational Research), Brussels Operational Center, Luxembourg, Luxembourg; 3 Aklilu Lemma Institute of Pathobiology, Addis Ababa University, Addis Ababa, Ethiopia; 4 National Tuberculosis Program (NTP), Conakry, Guinea; 5 Médecins Sans Frontières, Bo, Sierra Leone; 6 National Tuberculosis Control Program (NTCP), Ministry of Health, Manzini, Swaziland; 7 Faculty of Medicine-Gamal Abdel Nasser University, Conakry, Guinea; University of Cape Town, SOUTH AFRICA

## Abstract

**Setting:**

Ten targeted health facilities supported by Damien Foundation (a Belgian Non Governmental Organization) and the National Tuberculosis (TB) Program in Conakry, Guinea.

**Objectives:**

To uphold TB program performance during the Ebola outbreak in the presence of a package of pre-emptive additional measures geared at reinforcing the routine TB program, and ensuring Ebola infection control, health-workers safety and motivation.

**Design:**

A retrospective comparative cohort study of a TB program assessing the performance before (2013) and during the (2014) Ebola outbreak.

**Results:**

During the Ebola outbreak, all health facilities were maintained opened, there were no reported health-worker Ebola infections, drug stockouts or health staff absences.

Of 2,475 presumptive pulmonary TB cases, 13% were diagnosed with TB in both periods (160/1203 in 2013 and 163/1272 in 2014). For new TB, treatment success improved from 84% before to 87% during the Ebola outbreak (*P = 0*.*03*). Adjusted Hazard-ratios (AHR) for an unfavorable outcome was alwo lower during the Ebola outbreak, AHR = 0.8, 95% CI:0.7–0.9, *P = 0*.*04*). Treatment success improved for HIV co-infected patients (72% to 80%, *P<0*.*01)*. For retreatment patients, the proportion achieving treatment success was maintained (68% to 72%, *P = 0*.*05)*. Uptake of HIV-testing and Cotrimoxazole Preventive Treatment was maintained over 85%, and Anti-Retroviral Therapy uptake increased from 77% in 2013 to 86% in 2014 (*P*<*0*.*01*).

**Conclusion:**

Contingency planning and health system and worker support during the 2014 Ebola outbreak was associated with encouraging and sustained TB program performance. This is of relevance to future outbreaks.

## Introduction

Ebola Virus Disease (EVD)–commonly termed Ebola–is caused by Ebola viruses, and human-to-human transmission occurs through close contact with body fluids of infected patients. Case-fatality rates range from 30% [1] to 90% [2–4].

The recent Ebola outbreak in West Africa was identified in Guinea on March 21^st^ 2014 [5], and on August 8^th^ 2014 the World Health Organisation (WHO) declared the outbreak to be a “public health emergency of international concern”. To date, this is the largest and most sustained Ebola outbreak in history, with a total of 29,639 reported EVD cases and 11,316 deaths.

In Guinea, there were 3,804 EVD notified cases with 2,536 deaths (66%) [6]. Among notified cases there were 196 health-workers, 100 (51%) of whom died. Eighty one of these health-workers were from Conakry of which 37 died (46%) [7].

Guinea, located in West Africa, is bordered by Liberia and Sierra Leone. It has a population of about 12 million of whom about 2.4 million live in Conakry, the capital. There is a serious shortage of qualified health-workers with 0.1 physicians and 0.5 nurses and midwives per 1000 inhabitants [8].

The tuberculosis (TB) burden in Guinea is reflected by a prevalence of 253/100,000 habitants [9] (including 105 cases of multi-drug-resistant-TB) [9], and HIV prevalence among TB patients is 23% [10]. In addition, recurring disease epidemics are common, such as meningitis, cholera and measles [11].

Guinea, Sierra-Leone and Liberia were most affected by the Ebola outbreak. These countries had pre-existing health system limitations [12, 13], which were further exacerbated by the 2014 Ebola outbreak. Several health facilities were closed down temporarily or permanently due to underlying fear and distress among health-workers [14]–a problem compounded by inadequate training and lack of Ebola disinfection and protective material [15]. From a community perspective, fear and forced quarantine of Ebola-affected communities may also have negatively influenced health seeking behaviour and/or restricted movement of vulnerable individuals to health facilities [16, 17]. Other papers have highlighted serious gaps in access to maternal health services [18], reduced malaria consultations [19], lessened inpatient admissions and surgery [20], a decrease in new Tuberculosis (TB) diagnoses [21] and a decline in retention in HIV care [19, 21].

This underlying situation may have had a detrimental effect on the routine management of patients with TB, for whom adherence to treatment and health system resilience is key for achieving good TB program performance. For example, HIV-testing, which relies on finger-prick tests, may have dropped off because of health workers trying to minimise the risk of EVD transmission, and this in turn would have led to the reduced uptake of HIV-related interventions. Furthermore, since TB treatment in Guinea is centered on facility-based drug administration (observed pill swallowing and injectable administration), patients might have faced challenges receiving medication. Finally, drug stockouts could have occurred if routine drug procurement and supply activities were over-ridden by Ebola related priorities. All these factors could have negatively affected adherence to treatment, losses to follow up and survival probability.

During 2013, two medical doctors from Damien Foundation (a Belgian non-governmental organisation (NGO) which has been running a joint project with the National Tuberculosis Program (NTP) since 2007, conducted supervisory visits to health facilities accompanied by the district supervisor and NTP staff. Drug and consumable distributions were piggy-backed on such visits on a monthly basis. Tracing of patients who did not attend their scheduled visits was done by community-health workers. An initial phone call reminder was followed-up with a domiciliary visit using a motorcycle. All TB health-workers, including community-workers, received quarterly financial incentives.

During 2014, as part of pre-emptive action to cope with the Ebola outbreak challenges, the NTP of Guinea and Damien Foundation, introduced a package of additional measures. In brief, the package included: a) a joint decision by the NTP and Damien Foundation to keep open all health facilities involved with TB in Conakry, b) a “needs assessment” focused on infection control and health-worker safety, c) Ebola related training, d) quality control of laboratory services, e) monthly provision of consumables for prevention of Ebola, (f) screening for fever at health facility entry, and (g) enhanced support to health-workers through counseling about EVD, monthly on-the-job supervision and feedback on TB activities and performance [22].

We hypothesized that this package of additional measures was associated with sustained performance of the TB program during the Ebola outbreak. Our research question was: Has such support been useful for upholding TB performance during the Ebola storm? To answer this question we conducted a retrospective comparative cohort study involving ten targeted health facilities and compared TB performance between two periods: before (2013) the Ebola outbreak and during (2014) the outbreak when the additional package of measures was implemented. We also assessed whether the prolonged Ebola outbreak influenced TB management.

Specific objectives were to describe the accomplishment of additional support measures and to compare the following indicators for a period before (2013) and during (2014) the EVD outbreak:

1. Numbers of presumptive-TB cases and TB patients diagnosed, and number with any acquired Ebola infections.
2. Demographic and clinical characteristics of those starting TB treatment including uptake of HIV related interventions: HIV testing, Cotrimoxazole Preventive Treatment (CPT) and anti-retroviral treatment (ART).
3. TB treatment outcomes and survival probability stratified by new and retreatment cases of TB.
4. Drug stockouts, and
5. Presence of health-workers in TB services and number with any acquired Ebola infections.

## Methods

### Study design

This was a retrospective comparative cohort study.

### Setting

The study was conducted in Conakry which has five administrative districts. The health system in Conakry is pyramidal and includes 25 health centers, five district hospitals and two university hospitals [23]. The 25 health centers include 14 integrated TB diagnosis and treatment centers and 11 stand-alone treatment sites. Ten of these facilities were included as study sites: five integrated and five stand-alone centres. These ten facilities were selected because they had a TB notification rate of more than 150 TB cases per year, and a high TB prevalence (>200 TB cases/100,000 inhabitants).

### Study population and period

The study included all presumptive and diagnosed TB patients (new and retreatment) enrolled at the ten study sites between April and December 2013 (before the Ebola outbreak) and between the same months in 2014 (during the Ebola outbreak). TB patients referred-in from other TB centres (non study sites) during these two periods were also included. Patients with multidrug resistant TB were excluded.

2013 was deemed to be a suitable year for comparison based on the fact that the project under Damien Foundation and the NTP was well-established at this point and that there were no significant events (e.g. prolonged political unrest) during this year. The comparative months (April to December) were chosen on account of the start of the Ebola outbreak being officially declared at the end of March 2014.

We adhered to the Strengthening the Reporting of Observational studies in Epidemiology (STROBE) guidelines [24].

### Clinical management of TB

All TB patients are diagnosed and managed according to national guidelines [25, 26]. In brief, any presumed pulmonary TB patient provides two samples of sputum for microscopy; if one is positive the patient is diagnosed as pulmonary smear-positive. Pulmonary smear negative and extra-pulmonary patients are diagnosed by a medical officer at TB diagnosis and treatment centers. For extra-pulmonary TB other tests are performed such as radiography, biopsy, fluid analysis, etc. Sputum negative and extra-pulmonary TB are diagnosed in specialized health facilities not included as study sites. All diagnosed TB patients are offered HIV-testing, and those found to be HIV-positive are offered CPT and ART. They are then referred for TB treatment to any of the TB treatment centres closest to the patients’ residence.

During the 2014 Ebola outbreak all patients, including presumed and confirmed TB were screened for fever at entry to each health facility. Any patient presenting with unexplained haemorrhage, or with a fever or who had been in contact with a known Ebola case, or an ill or dead animal, was referred to the Ebola Treatment Center in Conakry [22].

TB-drug regimens and treatment outcomes are standardized and in line with NTP guidelines. New TB patients are treated for two months with fixed-dose combinations of rifampicin, isoniazid, pyrazinamid and ethambutol (RHZE), followed by four months with rifampicin and isoniazid (RH). Retreatment patients are treated during two months with streptomycin injectables and RHZE, followed by one month of RHZE and five months of RH and ethambutol fixed-dose combinations. TB management is free-of-charge to patients. Health facilities receive supplies on a monthly basis based on consumption.

#### Definitions of favorable treatment outcomes

All pulmonary smear positive patients that have a negative smear at the end of treatment are considered cured; if the patient completes treatment but there is no record of a negative smear, they are classified as treatment completed. Smear negative and extrapulmonary patients that finish treatment are classified as treatment completed. Treatment success is defined as cured and treatment completed.

#### Definitions of unfavorable treatment outcomes

Any TB case who stop treatment for more than two consecutive months is classified as lost to follow-up. Patients with a positive smear at five, six or eight months are considered treatment failures. Patients transferred to other health facilities to complete treatment are defined as transferred-out. Finally, death is recorded for those patients who die from any cause [26].

### Recording and Reporting System

The diagnosis and treatment centres are responsible for the elaboration of quarterly reports on TB case registration and treatment outcomes which include data from the treatment centres located on its area of responsibility. These reports are validated by the district supervisor, the NTP and Damien Foundation during supervisions visits at the end of each quarter.

#### Drug stockouts, presence of health-worker in TB services and Ebola infections among health-workers and TB patients

Drug stockouts were defined by the absence of one anti-TB drug (fixed-dose combinations and injectables) and the duration was expressed in number of days without an anti-TB drug.

The presence of health-workers in TB services was calculated on the basis of 6 working days per week (total of 216 days during each year of the study period) and the number of health-workers that should have been present and had been presented each day at the TB facilities (total of 38 health workers). This was calculated using the formula:

(Number of health workers) × (Work days actually present) ÷ (Number of Tb health workers) × (expected work days)

TB patients and Health-worker Ebola infections were recorded at the health facility and district level using standard notification forms.

#### Data collection, sources of data and validation

Following training, five dedicated data-collectors conducted data extraction between April and September 2015 using predesigned forms. They were supervised on a monthly basis and monitoring meetings organized. No identifying information on patients was collected. Patient master cards, treatment registers and laboratory registers were cross-checked for presumptive TB-cases, diagnosed TB and TB-HIV-cases and treatment outcomes. The health-worker attendance register, supervision reports and incentive payment registers were used and cross-checked to ascertain the presence of health-workers. Drug stockouts were assessed at each health facility using the pharmacy cards and quarterly TB reports. EVD infection among TB patients and health-workers was assessed using Ebola notification forms. All health-workers were obliged to notify Damien Foundation in case of illness (as per a formally agreed remuneration scheme) and additionally, there was a system in place for contact tracing of Ebola cases and identifying if the cases occurred in health workers. Data were double entered and validated using EpiData software (version 3.1, EpiData Association, Odense, Denmark).

### Sample size and statistical analysis

No sample size was calculated as all patients diagnosed and treated in the study sites were enrolled.

Differences between two groups (before and during Ebola outbreak) were assessed using the Chi-square test. Hazard Ratios (HR) per 100 person-years of follow-up were adjusted using a Cox-regression model. We analysed factors associated with favorable outcomes (defined as cure or treatment completed) and unfavourable outcomes (lost to follow-up, died, failure and transferred out) using univariable and multivariable Cox regression models adjusted for sex, age and TB category (new and retreatment). In addition, Kaplan-Meier survival probabilities without unfavourable outcomes were expressed graphically for new and retreatment TB patients and tested using the log-rank test. Data analysis was done using STATA (Version 11, Statacorp, USA).

### Ethics

Local ethics approval was obtained from the National Ethics Review Board of Guinea. The study also met the Médecins Sans Frontières (MSF) Ethics Review Board (Geneva, Switzerland) approved criteria for studies of routinely collected anonymised data and was also approved by the Ethics Advisory Group of the International Union Against Tuberculosis and Lung Disease (Paris, France).

## Results

The routine measures (Table 1) were reinforced with additional support measures summarized in Table 2.

**Table 1:** Accomplishment of routine Tuberculosis and Ebola related measures in ten health facilities supported by Damien Foundation and the National Tuberculosis Program before (April to December 2013) and during the Ebola outbreak (April to December 2014), Conakry, Guinea.

Measures	Year	2013	Year	2014
*Diagnosis*, *treatment and follow-up*	Programmed	Done	Programmed	Done
Supervision of health care facilities twice a week	180	145	180	160
		(80%)		(88%)
*Drugs and consumables*				
Monthly distribution of medicines and laboratory material	90	90	90	90
		(100%)		(100%)
*Refresher trainings*				
Quarterly one day refresher trainings on TB for health workers	3	3	3	3
		(100%)		(100%)
*Patient tracing and follow-up by community health workers*				
Number of patients traced	NA	495	NA	669
*Financial incentives*				
Quarterly payment of financial incentives to TB health workers	3	3	3	3
		(100%)		(100%)

TB = Tuberculosis; NA = Not applicable.

**Table 2:** Additional Tuberculosis and Ebola related support measures implemented by Damien Foundation and the National Tuberculosis Program during the Ebola outbreak (April to December 2014), Conakry, Guinea.

Measures	Year 2014
*General measures*:	
*Political will*	• Decision by Guinean NTP and Damien Foundation to sustain TB services during the Ebola outbreak
*Counseling support*	• Monthly feedback, enquiry and discussions about TB and Ebola related concerns among health workers
*Ebola specific measures*:	
*Needs assessment*	• Survey on infection control measures
*Training*	• Training of health workers on Ebola Virus Disease and infection control measures
*Consumables for infection prevention of Ebola*	• Monthly replenishment of Ebola protection and disinfection material at all health care facilities
*TB specific measures*:	
*Consolidation of one year safety-stock of TB and HIV drugs*	• Drugs purchased by the Guinean Government, the Global Fund, Damien Foundation, Deutsche Gesëllschaft für Internationale Zusammenarbeit (GIZ), MSF and the Ordre de Malta
*Quality control of Laboratory services*	• Quarterly External Quality Control Assessment
*Patient tracing and telephone follow-up by health-workers*	• Telephone reminders for those patients not turning up for scheduled follow-up visits (Refund of telephone bills)
*Financial incentives*	• Increase in the financial incentive package during the Ebola outbreak
*Feedback*	• Monthly feedback on TB activities and quarterly feedback on TB reports to the head of each health facility

TB = Tuberculosis; NTP = National Tuberculosis Program

**Fig 1:**
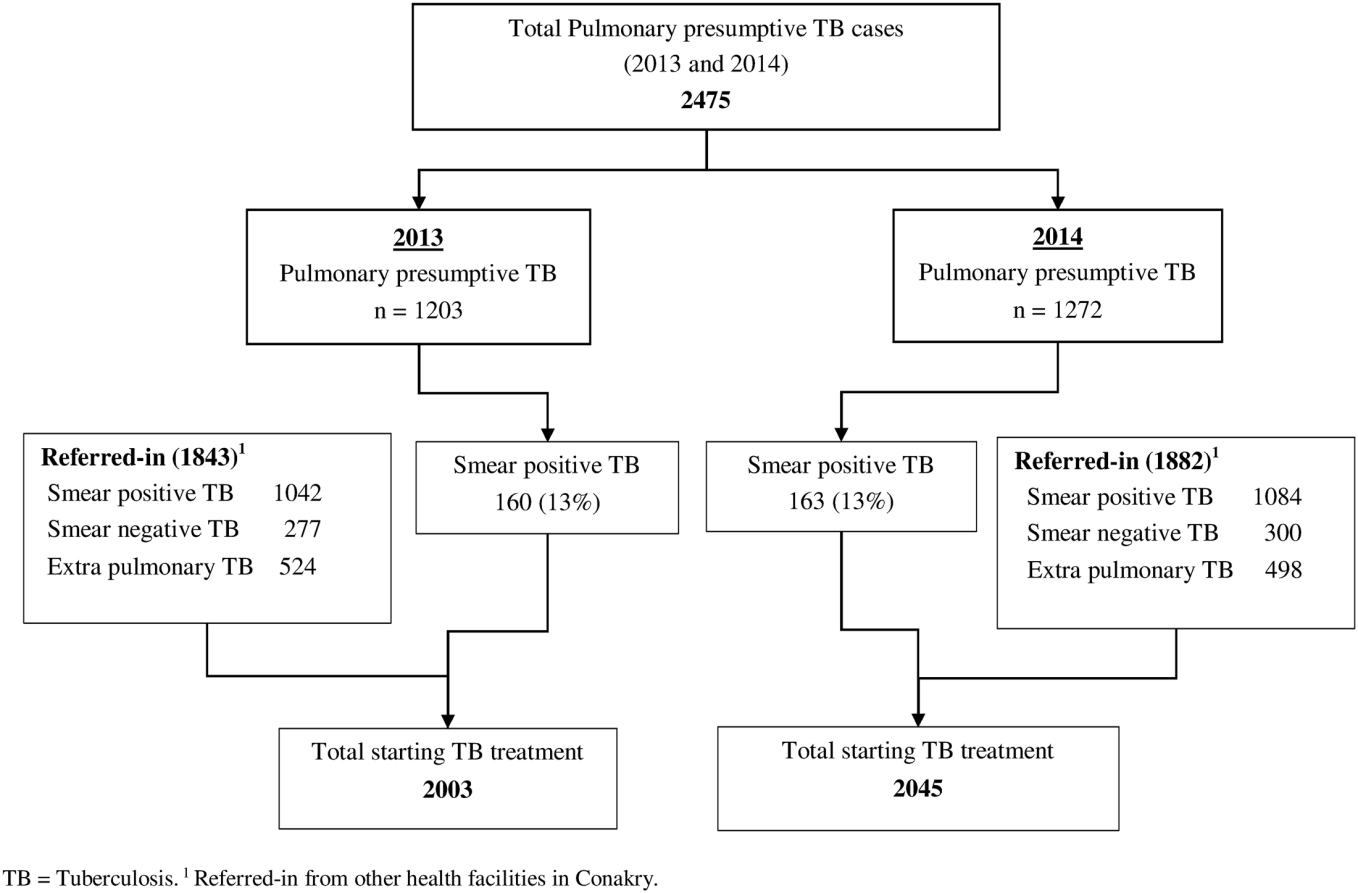
Pulmonary presumptive TB cases diagnosed with TB and referred in patients in ten health facilities supported by Damien Foundation, before (April to December 2013) and during the Ebola outbreak (April to December 2014), Conakry, Guinea. This figure shows presumptive-TB cases, those diagnosed with TB and those referred-in for treatment.

The proportion of smear positive TB patients diagnosed (13%) among presumptive cases and overall number of inward referrals was similar during the two periods (Fig 1).

### Patient characteristics and uptake of HIV-interventions

No acquired Ebola infections were notified among TB patients. The patient characteristics and uptake of HIV-interventions among TB patients was similar in both periods. Similarly, HIV-testing and CPT uptake was maintained over 85%, HIV positivity among TB patients tested was 18% in both periods, and ART uptake increased from 77% in 2013 before the Ebola outbreak to 86% in 2014 during the Ebola outbreak (*P =* 0.001), (Table 3).

**Table 3:** Patient characteristics including uptake of HIV-interventions among tuberculosis patients in ten health facilities supported by Damien Foundation, before (April to December 2013) and during the Ebola outbreak (April to December 2014), Conakry, Guinea.

	Before Ebola	During Ebola	*P-value
	n(%)	n(%)	
Total	2003	2045	
**Sex**	798(40)	779(38)	
Females			
Males	1205(60)	1266(62)	0.3
**Age (years)**			
<35	1334(67)	1354(66)	
≥35	669(33)	691(34)	0.8
**TB type**			
Smear positive	1202(60)	1247(61)	
Smear negative^1^	277(14)	300(15)	0.4
EPTB	524(26)	498(24)	0.2
**TB Category**			
New	1896(95)	1934(95)	
Retreatment	107(5)	111(5)	0.9
**Treatment regimen**			
Short course	1859(92)	1890(92)	
Paediatric	45(2)	44(2)	0.8
Retreatment	107(6)	111(6)	0.9
**HIV related interventions**			
Known HIV positive	47(2)	84(4)	
Eligible for HIV test	1956	1961	
HIV tested	1739(87)	1867(91)	<0.001
Of whom:	314(18)	321(18)	0.5
HIV positive			
On CPT	276(88)	312(97)	<0.001
On ART	242(77)	278(86)	0.001

* Uncorrected chi square comparing proportions before and during Ebola outbreak.

^1^ Including Sputum not done for five patients in each group;

TB = Tuberculosis; EPTB = Extra Pulmonary Tuberculosis; HIV = Human Immunodeficiency Virus; CPT = Cotrimoxazole Preventive Therapy; ART = Anti Retroviral Treatment.

### TB treatment outcomes and survival probability stratified by TB category

Table 4 shows TB treatment outcomes. For all new TB cases, treatment success was 84% before and 87% during the Ebola outbreak (*P =* 0.03). Among HIV-co-infected patients, treatment success was higher and deaths fewer during the Ebola outbreak than before. For retreatment TB, treatment success was 68% before and 72% after the Ebola outbreak.

**Table 4:** Tuberculosis treatment outcomes among tuberculosis patients placed on treatment in ten health facilities supported by Damien Foundation, before (April to December 2013) and during the Ebola outbreak (April to December 2014), Conakry, Guinea.

	Before Ebola	During Ebola	*P-value
TB treatment outcome	n(%)	n(%)	
**±New TB (all types)**			
**Total**	**1896**	**1934**	
Treatment success	1598(84)	1672(87)	0.03
Dead	142(8)	117(6)	0.04
Failure	17(1)	26(1)	0.2
Transfer out	76(4)	57(3)	0.07
Lost-to-follow-up	63(3)	62(3)	0.8
**Co-infected HIV/TB			
**Total**	361	405	
Treatment success	259(72)	324(80)	0.007
Dead	72(20)	51(13)	0.005
Failure	3(1)	5(1)	0.6
Transfer out	18(5)	8(2)	0.02
Lost-to-follow-up	9(2)	17(4)	0.3
**±Retreatment TB**			
**Total**	107	111	
Treatment success	73(68)	80(72)	0.5
Dead	12(11)	16(14)	0.5
Failure	2(2)	1(1)	0.6
Transfer out	7(7)	5(5)	0.5
Lost-to-follow-up	13(12)	9(8)	0.2

* Uncorrected chi square comparing proportions before and during Ebola outbreak; ± Including HIV/TB patients.

** Including, new and retreatment cases;

TB = Tuberculosis. HIV = Human Immunodeficiency. PTB = Pulmonary TB.

**Fig 2:**
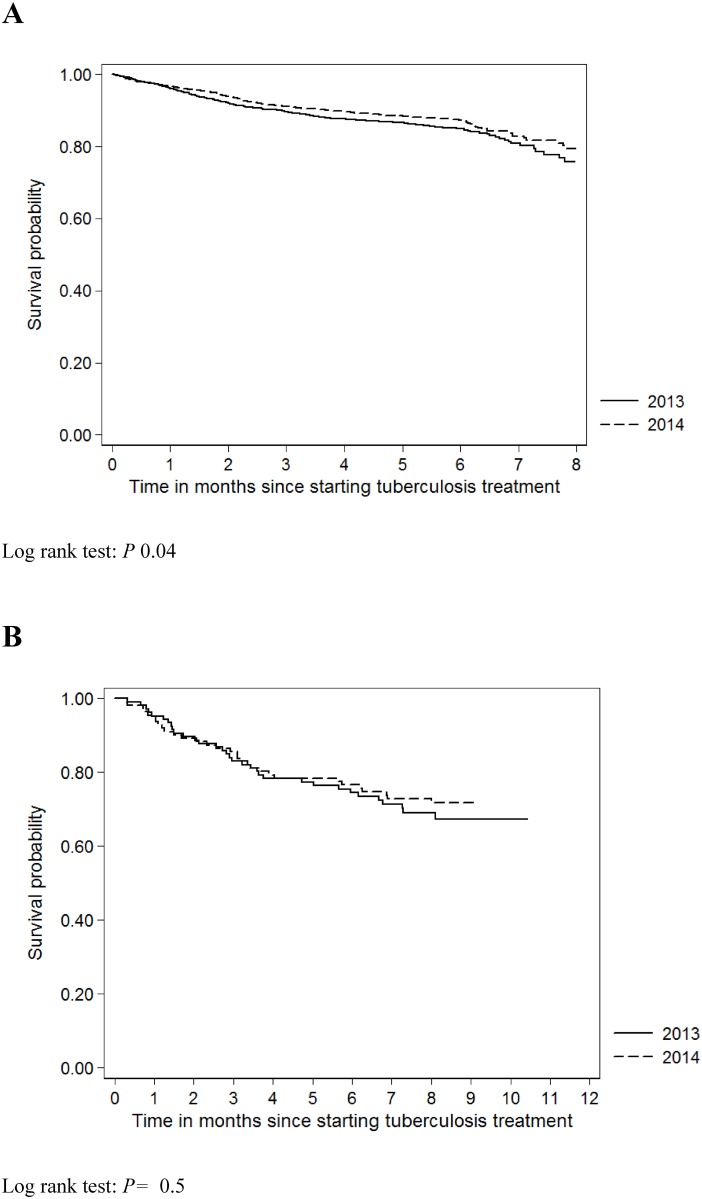
Survival probability of Tuberculosis new (A) and retreatment (B) cases placed on treatment in ten health facilities supported by Damien Foundation, before (April to December 2013) and during the Ebola outbreak (April to December 2014), Conakry, Guinea. This figure shows the probability of having an unfavourable outcome (lost to follow-up, treatment failure, death or transferred out) during the Ebola outbreak (2014) compared to the periode before (2013).

The probability of having an unfavourable outcome (lost to follow-up, treatment failure, death or transferred out) during the Ebola outbreak (2014) compared to the periode before (2013) was statistically significant inferior for new TB cases, but it was not statistically different for retreatment TB (Fig 2).

The risk of an unfavourable outcome (lost to follow-up, treatment failure, death or transferred out) was lower among new TB cases during the 2014 Ebola outbreak compared to the year before the outbreak (Adjusted Hazard-ratio 0.8, 95% CI: 0.7–0.9, *P = 0*.*04*, Table 5).

**Table 5:** Factors associated with *unfavorable outcomes among tuberculosis patients placed on treatment in ten health facilities supported by Damien Foundation, before (April to December 2013) and during the Ebola outbreak (April to December 2014), Conakry, Guinea.

	Univariate		Multivariable	
Variables	HR(CI 95%)	*P*-value	AHR(CI 95%)	*P*-value
**±New TB (all types)**				
Year of enrolment				
2013	1		1	
2014	0.8(0.7–1)	0.05	0.8(0.7–0.9)	0.04
Sex			1	
Female	1			
Male	1.1(0.9–1.3)	0.29	1.1(0.9–1.2)	0.37
Age (years)				
< 34	1		1	
≥ 35	1.6(1.4–1.9)	< 0.001	1.6(1.4–1.9)	< 0.001
**±Retreatment TB**				
Year of enrolment				
2013	1		1	
2014	0.8(0.5–1.4)	0.5	0.9(0.5–1.5)	0.8
Sex				
Male	1		1	
Female	1.3(0.7–2.3)	0.3	1.3(0.7–2.4)	0.2
Age (years)				
< 34	1		1	
≥ 35	1.2(0.7–2.1)	0.3	1.3(0.8–2.1)	0.2

* Unfavorable outcomes = lost-to follow-up, failure, died & transfer out. ± Including HIV/TB patients.

HR = Hazard Ratio, CI = Confidence Interval at 95%, AHR = Adjusted Hazard Ratio, TB = Tuberculosis.

### Drug stockouts

For fixed-dose combination TB drugs, there were five episodes of stockouts in 2013, but none for injectables; the median stockout time was 5 days (range 2–8 days). There were no episodes of TB drug stockouts in 2014.

### Presence of health workers in TB Services and acquired Ebola infections

On the basis of 6 working days per week and a total of 216 expected work days health-worker availability for 38 health-workers was 98% before the Ebola outbreak, and 99% during the Ebola outbreak (a total absence of 4.3 days in 2013 and 2.16 days in 2014 in each health facility). During absences from a given health facility, at least one health-worker was always available to ensure daily treatment and follow-up activities. There were no acquired Ebola infections among health facility and community-TB workers in any of the ten health facilities. One death of a physician was reported in 2014, but was unrelated to Ebola.

## Discussion

This is the first study from West Africa assessing the effect of the 2014 Ebola outbreak on TB program performance in the presence of an additional support package to cope with Ebola. It showed that in the presence of enhanced health systems support to uphold TB services, TB program activities and performance were sustained, all TB facilities remained open and importantly, no health workers were infected with Ebola.

There were also no stockouts and health worker availability was sustained at 98% during the entire study period. This is encouraging and in stark contrast to other health settings in Guinea, Sierra Leone and Liberia where the story was one of general turmoil: steep drops in diagnosis of TB and HIV [21], reduced retention of patients in care [27], stockouts and rampant health facility closures due to the occupational risk of contracting Ebola [28].

These findings support the added value of pre-emptive action aimed at bolstering a fragile health system in a country where epidemics are frequent and disruptive, and resources for healthcare are limited. In this light, the crucial and complementary role played by a well-resourced NGO throughout the Ebola outbreak is commendable. This experience also highlights the potential role and relevance of such support for other diseases control programs in West Africa. Importantly, in Guinea, such support depended on the strong partnership between the NTP and Damien Foundation and the availability of committed human resources regularly supported by an additional package of measures. These additional inputs were covered by Damien Foundation and were included in the annual project budget of €300,000.

The study strengths are that the additional support measures for Ebola were introduced within the routine framework of the NTP and are thus replicable; data on Ebola infections, presence of health workers, TB treatment outcomes were rigorously gathered from all health facilities and there were no missing outcomes. Despite the retrospective nature of the study, the upfront supervision of data collectors, monthly follow up meetings and correction of errors should have contributed to data robustness. The data were double-entered by dedicated data entry clerks and validated. Finally, this study responds to a recent call for pre-emptive action and operational research on Ebola and TB [29, 30].

A main study limitation is that we did not have a control group of health facilities and as such are unable to attribute a cause-effect relationship. This notwithstanding, we feel it is logical and intuitive to assume that the additional package geared towards reinforcing the routine TB program, ensuring Ebola infection control, health worker safety and improving motivation would have had a beneficial effect. This thinking is further supported by other studies from Guinea and Liberia showing that, without focused attention on these aspects, health facilities were often abandoned and utilisation of HIV, malaria and reproductive health services were seriously compromised [18, 19, 21, 27]. For example, in Liberia 60% of all HIV clinics were closed down due to fear and lack of health-worker safety [27].

In Guinea a study among patients on ART, showed that the loss-to-follow-up escalated from 0 to 42% [31].

In another study in Macenta, Guinea, where the impact of Ebola on HIV and TB services was assessed, HIV-testing dropped by 40% compared to the pre-outbreak period. Similarly new TB diagnoses declined by 53% and enrolment in HIV care dropped by 47% [21]. This could be explained by the fact that Macenta is located at the epicenter of the Ebola outbreak and was one of the most affected regions. Patients from there and other affected regions arrived in Conakry seeking health care. This may explain why the number of cases referred-in fluctuated in the middle months of the EVD outbreak compared to the respective months of 2013 (before the outbreak).

The study findings have a number of important policy and practice implications. First, the presence of a well-resourced NGO working in close collaboration with the NTP and partners can facilitate implementation on the ground. Understandably, when existing health system resources are overwhelmed by emergencies or epidemics, such an “added hand” seems to bear dividends.

Second, surprisingly, there were higher uptakes of all HIV-related interventions and better TB treatment outcomes during the Ebola outbreak. CPT and ART uptake significantly improved during the outbreak, as did treatment success for HIV co-infected patients. Impressively, among new TB cases treatment success even crossed the desired WHO threshold of 85%.

Training on Ebola and infection prevention, counselling about Ebola related concerns were also important to encourage health-workers to continue working.

We do not know the exact reasons for these findings, but suspect that they can be attributed, in part at least, to the enhanced package of support measures (the Quality control and procedures and regular feedback on laboratory performance, measures that encouraged continued health worker presence–training on Ebola and infection control, counselling, financial incentives etc.), along with surplus stocks of TB drugs, CPT and ART made available at the end of 2013 (prior to the outbreak). We believe additional drug availability not only served as a “safety-net” for preventing drug stock interruptions but also improved medication access. A lesson learnt is that in Ebola-prone countries of West Africa, and otherwise fragile health systems, it is worthwhile to maintain a “safety-stock” of medication for TB and HIV at any time.

In addition, involvement of the head of TB health facilities, enforced follow up of TB patients through phone call reminders and domiciliary visits with the community health workers, may have been beneficial during the Ebola outbreak. Finally, community involvement in TB activities has been endorsed since Damien Foundation partnered up with the NTP in 2007 and this has likely contributed to community confidence and acceptability of TB services in Conakry.

Third, a critical question is why the ten TB health facilities in Conakry were “safe havens” in terms of occupational risk for Ebola among TB patients and health-workers. These facilities also offered general health services and were thus prone (like all others) to the occupational risk for Ebola, and yet we had no acquired Ebola infections among health workers. This is in stark contrast to other health facilities in Conakry, where there were 81 health-worker infections and 37 deaths. This scenario is likely linked to the additional support package provided during the Ebola outbreak under the coordination of the NTP and Damien Foundation. In brief, all health workers within TB services were involved in Ebola surveillance, the frequency of supervision was increased and there was a strong focus on training and support of health workers particularly in relation to infection prevention and counseling for any Ebola related concerns. This not only served to safeguard their occupational health, most importantly, but also to encourage their continued presence at the health facilities. Adequate human resource capacity is essential for upholding a minimum delivery and quality of care, including adherence to necessary infection control measures. Finally, given Damien Foundation’s long term approach, a pre-established level of confidence among staff may have played a role here.

Keeping health facilities safe is of high relevance to TB control and also enhances the much needed interaction between disease-specific programmes and health systems [32]. We thus reiterate our call to the WHO and governments of West African countries to establish dedicated umbrella units which would focus upon and guide health-worker and facility safety (i.e. occupational health) [30].

In conclusion, contingency planning and health system and health-worker support during the 2014 Ebola storm was associated with encouraging and sustained TB program performance. This is of relevance not only to global public health but also to the post 2015 End TB Strategy–a plan to end the Global TB epidemic [33].

## Supporting Information

S1 File Health staff Data Set. **S1_File** contains the database about health workers availability at health facilities.
(DTA)

S2 File Tuberculosis patients Data Set. **S2_File** contains the database about tuberculosis patients.
(DTA)

S3 File Stock of tuberculosis drugs Data Set. **S3_File** contains the database about stockouts of tuberculosis drugs.
(DTA)

S4 File Tuberculosis presumptive cases Data Set. **S4_File** contains the database about presumptive cases of tuberculosis.
(DTA)
